# Structural Neuroimaging in the Detection and Prognosis of Pre-Clinical and Early AD

**DOI:** 10.3233/BEN-2009-0230

**Published:** 2009-10-21

**Authors:** Christine Fennema-Notestine, Linda K. McEvoy, Donald J. Hagler, Mark W. Jacobson, Anders M. Dale, the Alzheimer’s Disease Neuroimaging Initiative

**Affiliations:** ^1^Deptartment of PsychiatryUniversity of California San DiegoSan DiegoCAUSA; ^2^Department of RadiologyUniversity of California San DiegoSan DiegoCAUSA; ^3^Veterans Affairs San Diego Healthcare SystemSan DiegoCAUSA; ^4^Department of NeurosciencesUniversity of California San DiegoSan DiegoCAUSA

**Keywords:** MRI, Alzheimer’s disease, Mild Cognitive Impairment (MCI), morphometry, brain imaging

## Abstract

Current research supports the strong potential of structural MRI profiles, even within cross-sectional designs, as a promising method for the discrimination of Alzheimer's Disease (AD) from normal controls and for the prediction of Mild Cognitive Impairment (MCI) progression and conversion to AD. Findings suggest that measures of structural change in mesial and lateral temporal, cingulate, parietal and midfrontal areas may facilitate the assessment of a treatment's ability to halt the progressive structural loss that accompanies clinical decline in MCI. The performance of prediction is likely to continue to improve with the incorporation of measures from other neuroimaging modalities, clinical assessments, and neuromedical biomarkers, as the regional profile of individuals at risk for progression is refined.

## 1. Introduction

As one of the most serious health issues facing the U.S. today, AD may afflict an estimated 13.2 million people in 2050, with current cost of caring for individuals with AD estimated at $148 billion per year in the US [1,12]. Millions more individuals are suffering prodromal cognitive impairment that may be related to AD [40]. To facilitate the development of therapies aimed at preventing or delaying the progression of AD, research has focused on the search for sensitive, non-invasive, *in vivo* biomarkers that would enable earlier, more accurate clinical diagnosis and aid in monitoring disease progression and the effectiveness of therapeutic intervention [26,35,36,82]. Neuroimaging measures, which are sensitive to AD-related neurodegeneration early in the disease, hold significant promise as direct measures of disease-modifying treatment effects [66,73,82]. Numerous neuroimaging approaches provide insight into the neuronal and synaptic loss of AD, including structural MRI, positron emission tomography (PET), and functional MRI. Such measures may improve the sensitivity and specificity of detecting AD at early stages and assist in identifying individuals at risk for developing AD. Early identification would allow treatment to begin before significant functional impairment or extensive irreversible neuronal damage occurs and may permit shorter treatment trials by focusing on patients with high likelihood of imminent conversion. Structural MRI measures in particular are non-invasive and relatively free from confounding behavioral factors, and, as a result, these measures may be less variable in assessing treatment effects relative

*Corresponding author: Christine Fennema-Notestine, Ph.D., UCSD School of Medicine, 9500 Gilman Dr #0841, La Jolla, CA 92093-0841, USA. Tel.: +1 858 822 3198; Fax: +1 858 534 1078; E-mail: Fennema@UCSD.edu.
[1]This review emphasizes findings based on data obtained from the Alzheimer's Disease Neuroimaging Initiative (ADNI) database (www.loni.ucla.edu\ADNI). As such, the investigators within the ADNI contributed to the design and implementation of ADNI and/or provided data but did not participate in analysis or writing of this review. Complete listing of ADNI investigators available at http://www.loni.ucla.edu/ADNI/Data/ADNI_Authorship_List.pdf.

ISSN 0953-4180/09/$17.00 © 2009 – IOS Press and the authors. All rights reserved to performance on commonly used cognitive tests [46, 48]. This decrease in variability may result in greater statistical power in clinical trials, enabling the use of smaller sample sizes. Cross-sectional, structural MRI research has provided insight into the neuroanatomical profiles of pre-clinical and early AD and has demonstrated significant value in the prediction of conversion and disease progression.

## 2. Neuropathology of Alzheimer's disease

Neuropathological studies have defined the basic neurodegenerative profile of AD through the distribution of hallmark features, amyloid plaques and neurofibrillary tangles. While amyloid deposits in the cerebral cortex show considerable inter-individual variation in density and distribution in the early stages of AD, the characteristic distribution of neurofibrillary pathology allows post-mortem differentiation of progressive stages of AD [9,10]. Neurofibrillary changes first appear in the transentorhinal area, then migrate into limbic areas before spreading into neocortical association areas [9,10,66]. Clinical diagnosis of AD typically accompanies the third stage, when modest neuropathology is observed in hippocampal structures and severe neuropathology is observed in the transentorhinal and entorhinal areas, affecting many of the projection neurons within these areas [9,10]. Structural MRI has provided insight into identifying the profile of early stage pathogenesis *in vivo*.

## 3. Mild cognitive impairment

The study of individuals with Mild Cognitive Impairment (MCI), which has been recognized as a potential prodromal stage of AD, may increase our ability to detect early changes. MCI refers to a syndrome of impairment on one or more standardized tests of cognitive function of insufficient severity to cause functional impairment [64,66,68,69]. When memory is one of the cognitive domains involved, often referred to as "amnestic MCI," individuals are at increased risk of converting to AD, estimated at 10–15% per year compared with 1–2% per year for cognitively intact elderly [68]. In addition, at autopsy the majority of amnestic MCI individuals show pathological features of AD, supporting amnestic MCI as a transitional state between healthy aging and AD [60,64,70]. MCI is a heterogeneous disorder, however, as some individuals do not progress, others convert to non-AD dementia, and a few revert to normal cognitive status [8]. Autopsy studies of amnestic MCI cases have confirmed this heterogeneity with individual cases demonstrating non-AD pathology, such as hippocampal sclerosis or subcortical ischemic vascular disease, despite similar cognitive characterization [49,70]. There is significant potential for neuroimaging to provide a more comprehensive characterization of AD-related structural changes in MCI individuals that may further define the prodromal stages of AD.

## 4. Cross-sectional structural MRI in AD and MCI

As expected from the neuropathological and clinical profiles of AD, structural MRI has supported significant atrophy in mesial temporal structures affected early in the neurodegenerative process as well as in temporoparietal association areas [3,9,10,71,77,83,84]. These studies in AD individuals have also shown that early atrophy in these regions is followed by more extensive involvement of frontal and parietal regions, leaving primary and secondary sensory areas relatively spared until late in the disease [3,9,10,77,83,84]. Consequently, studies in early AD and in those at risk for AD, such as individuals with MCI, have focused primarily on mesial temporal regions, demonstrating that hippocampal and entorhinal cortex typically are smaller than those measured in controls [3,5,7,14,24,45,52,53,58,76,88], predictive of future conversion to AD [18,19,21,44,47,58, 59,79], and correlated with memory impairment [25, 37]. The definitive classification and specificity of these changes to AD, particularly for the hippocampus, remain a challenge [85], and ongoing work continues to examine additional regions and patterns of regional changes [42,54,56,75,76,84,87]. The cingulate cortex, for example, has been shown to exhibit changes in at-risk individuals [41,50] and in those known to eventually convert to AD [57], and recent work suggests that MCI individuals with impairment in other cognitive domains in addition to memory (multiple-domain MCI, or MMCI) may evidence thinner precuneus cortices relative to individuals with impairments restricted to the memory domain (single domain MCI, or SMCI) [75]. An accurate depiction of the earliest patterns of degenerative changes will require extensive studies of individuals in both preclinical and prodromal stages of AD.

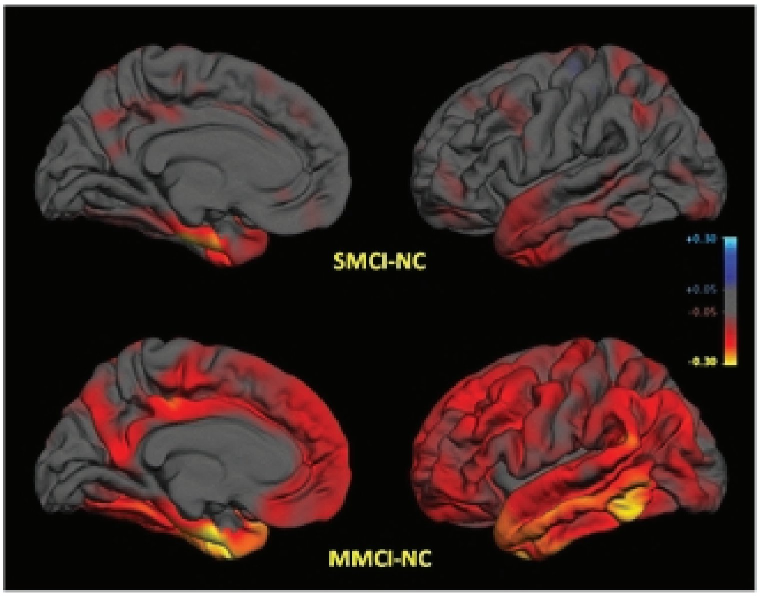

Fig. 1. Group differences in average thickness (mm) for left hemisphere. *Top row*: SMCI vs. NC; *Bottom row*: MMCI vs. NC. *LEFT* mesial views, *RIGHT* lateral views. The scale ranges from $<$–0.3 (yellow) to $> +$ 0.3 (cyan) mm thickness. Areas on the red-yellow spectrum indicate regions of thinning with disease: approximate color scale in mm is $-0.05$ to $-0.15$ dark red, $-0.20$ bright red, $-0.25$ orange, and $< -0.30$ yellow. Any differences smaller than $+/-$ 0.05 mm are gray.

## 5. Neuroanatomical profiles

In this context, our recent work has emphasized the role of cross-sectional, structural MRI in providing neuroanatomical profiles of pre-clinical and early AD and in the prediction of disease progression [29, 61]. Our studies are based on data publicly available from the multi-site Alzheimer's Disease Neuroimaging Initiative (ADNI), an important effort designed to facilitate the scientific evaluation of neuroimaging and other biomarkers in the onset and progression of MCI and AD [43,65,66]. Using methods developed within the NIH/NCRR sponsored Morphometry Biomedical Informatics Research Network (mBIRN) and the ADNI [30,31,33,39,43,51], we studied normal elderly participants and individuals with MCI and AD. The MCI participants were impaired on one or more standardized tests of cognitive function, with memory as one of the impaired domains (i.e., amnestic MCI); demonstrated no associated functional impairment; and did not meet criteria for clinical diagnosis of dementia at the time of these baseline scans [68]. In addition, during our morphometric characterization, we defined neuropsychological subgroups of the MCI cohort, dividing the group into those with SMCI, which may reflect the earliest stage of prodromal AD, and those with MMCI, which may represent a later disease state.

## 6. Characterization of single-domain, amnestic MCI

We characterized the MCI ($n = 175$) and AD ($n =$ 84) cohorts relative to the normal controls ($n = 139$) using methods based on volumetric segmentation [30, 31] and cortical surface reconstruction and parcellation [15,16,20,32,33] techniques for each individual's brain, combining region of interest (ROI) and cortical surface vertex-wide approaches that allow greater exploitation of information obtained from all brain areas [29]. The results demonstrated robust performance of the methods and evidenced measurements sufficiently sensitive to reveal the subtle morphometric characterization of SMCI and MMCI in this cross-sectional sample of individuals who may be in a prodromal AD state (Fig. 1). While significant mesial temporal atrophy characterized all groups, including SMCI, MMCI, and AD, smaller volumes and thinner cortex were widespread and evident across the cortex even within the SMCI group.

The regions that were most sensitive to early changes were within temporal, rostral posterior cingulate, inferior parietal, precuneus, and caudal midfrontal cortices (Fig. 1). Areas critical for distinguishing SMCI from NC were in the mesial temporal regions as expected, including bilateral hippocampus, bilateral entorhinal cor-

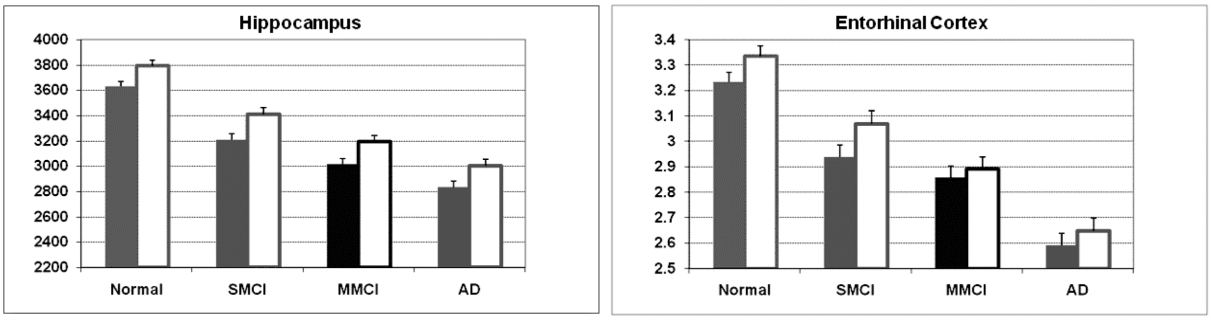

Fig. 2. Estimated marginal mean volume (mm3) for Hippocampus and Entorhinal Cortex thickness (mm) by group accounting for sex and age effects. *Error bars* = std error of the mean. *Solid bar* = Left Hemisphere; *outlined open bar* = Right Hemisphere.

tex, and left amygdala, similar to recent findings [42, 75,76,87], supporting these regions as the most sensitive to early AD-related changes. Given previous work suggesting that mesial temporal asymmetries also may be modified by disease state, we also examined left/right asymmetries as potential measures indicative of risk or disease progression. Building on findings in AD (e.g. [4,55,83]), several investigators [75,78] have suggested that left hemisphere regions may be more affected than the right, changing the natural asymmetries, in individuals at increased risk for AD, although reports are conflicting [56]. Our findings supported natural right dominant asymmetries in the hippocampus and entorhinal cortex of normal controls, as suggested in some previous work (e.g. [28,67]), however, these asymmetries did not interact with diagnostic group providing no support for structural asymmetries as an indication of disease state (Fig. 2).

Importantly, in the SMCI cohort, characterized with impairment in the memory domain only, significant atrophy extended beyond the mesial temporal regions into posterior cingulate, parietal, and frontal regions indicating that widespread damage occurs before standard clinical measures can detect AD. The pattern of thinning in SMCI overlapped to some extent with recent findings by Seo and colleagues [75], although our larger sample demonstrated greater thinning in medial parietal, lateral middle temporal, and anterior cingulate regions and powered significant differences from NC in numerous ROIs. These broad changes even within individuals impaired only in the memory domain suggest that standard neuropsychological measures may not be sufficiently sensitive, although novel approaches may be more informative (see Jacobson et al., this issue). The pattern of results also broadly supported the view that MMCI may be a later stage of prodromal AD than SMCI, although some of the structural changes observed confirmed the potential heterogeneity of the sample. Relative to SMCI, the presumably later stage of MMCI showed greater atrophy in the inferior and lateral temporal lobe, and significantly thinner temporo-parietal association cortices, retrosplenial, anterior cingulate, and other frontal regions; these significant findings were more widespread relative to previous work [75], perhaps due to a larger MMCI sample. The AD and MMCI groups were relatively similar, with greater differences in some parietal, retrosplenial and frontal regions. These profiles may prove useful in defining a pattern of degeneration that will enable discrimination of early stage AD from other disorders that also impact mesial temporal regions.

## 7. Proposed trajectory based on cross-sectional data

In this cross-sectional sample, we further explored the potential “progression” from NC to AD employing regression models assessing the linear and quadratic effects. As expected, the earliest and most dramatic effects across groups were evident within the entorhinal cortex. Rostral posterior cingulate was reduced, similarly across groups, followed by additional temporal, parietal, and frontal regions. Of the later changing regions, the lateral middle temporal gyrus may change most rapidly, followed by the posterior cingulate and inferior parietal cortices, and then the rostral middle frontal region. The proposed sequence of change supports neuropathological findings as degeneration begins in the mesial temporal area, moves through rostral posterior cingulate and medial orbitofrontal cortices, followed by lateral temporal, retrosplenial and inferior parietal regions, and, subsequently, mid frontal cortex. The growing body of evidence thus suggests that there is significant, widespread cortical atrophy in preclinical stages of AD, including areas outside the mesial temporal lobe. These cross-sectional findings then lead to the question of whether changes in these regions are predictive of conversion to AD.

## 8. Discrimination, classification, and prediction

Methods to facilitate the prediction of which MCI individuals are likely to convert to AD could increase efficiency of clinical treatment trials by allowing them to focus on individuals with high likelihood of imminent conversion, permitting shorter follow-up periods to demonstrate efficacy, alongside a potential utility for individual clinical prognosis. While previous work has supported the predictive power of mesial temporal regions for future conversion to AD [2,18,19,21, 23,44,47,58,59,79], these more extensive morphometric findings in MCI may increase the power to predict conversion to AD. Cross-sectional differences in hippocampal and entorhinal volumes have been found to discriminate between MCI patients who convert to AD and those who remain stable [21,47,58,79]. Cross-sectional differences in other brain areas, such as the amygdala [19] or the volume of the temporal horn of the lateral ventricle [59], have also been shown to predict conversion to AD. However, these classifiers may have limited specificity to AD [47].

In this light, combinations of regional structural measures may increase accuracy in predicting imminent risk of conversion to AD. Our recent work [61] employed multivariate procedures to capitalize on this morphometric signature associated with early AD in the prediction of disease progression in MCI, potentially increasing both specificity and sensitivity in detecting AD at early stages. Several studies have shown improvement in classification accuracy when more than one measure is used, often combining hippocampus and an additional measure such as the amygdala or posterior cingulate volumes [11,38,47,80]. Additional small studies have approached the search for a predictive structural profile through voxel-based techniques, such as density maps and voxel based morphometry [17, 81]. In a study of MCI and control individuals, a high-dimensional pattern classification technique resulted in a cross-validated accuracy of 90% for discriminating 15 MCI from 15 matched controls [17]. The resulting classification algorithm included areas within the lateral and inferior regions of the hippocampus, and regions in bilateral superior, middle, and inferior temporal gyri, bilateral orbitofrontal, left fusiform and posterior cingulate. Another study found that although the voxel-based approach resulted in superior classification relative to hippocampal volume alone, the combination of the two provided the best classification [81]. These studies demonstrate the promise of employing patterns of structural information in classification, although the reliability of these approaches in larger samples with individuals further from conversion to AD should be examined. The potential for neuroimaging biomarkers to be predictive of conversion risk based on a baseline assessment for individuals with MCI may prove to be more advantageous.

This issue is presently under investigation by several groups using baseline data from the ADNI. Fan and colleagues [27] analyzed baseline MRI data from 66 controls, 88 individuals with MCI, and 56 individuals with AD from the ADNI. High dimensional pattern classification methods were applied to voxel based morphometric measures from the healthy controls and AD subjects to identify a pattern of atrophy characteristic of AD. The resulting pattern was spatially complex, involving widespread brain regions. Importantly, individuals with MCI who displayed this pattern showed greater 1-year clinical decline than those who did not [27].

## 9. Discrimination of controls and AD

We have recently extended these findings by applying multivariate procedures to a larger subset of the ADNI cohort (the ADNI sample characterized earlier [29], which included 139 controls, 175 individuals with MCI, and 84 with AD) [61]. We were able to identify a pattern of regional atrophy characteristic of AD and to demonstrate that the presence of this pattern in individuals with MCI was predictive of 1-year clinical decline and structural volume loss [61]. With stepwise linear discriminant analysis (LDA) using candidate input morphometric variables from 58 ROIs, including lateral ventricles, mesial temporal structures, and cortical association areas, we identified a pattern of regional atrophy that best discriminated AD from NC. The regional pattern that best discriminated NC from AD subjects involved eight measures, including left hippocampal volume and cortical thickness of right entorhinal, right middle temporal, left bank of the superior temporal sulcus, right retrosplenial, right superior temporal, left medial orbital frontal, and right lateral orbital frontal regions.

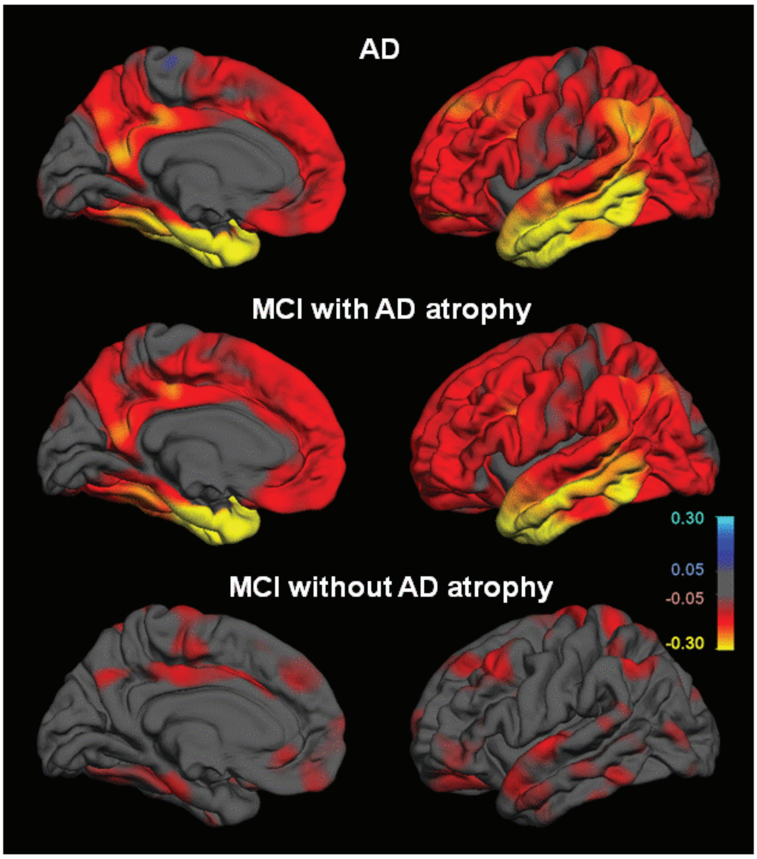

Fig. 3. Group differences in average thickness (mm) for left hemisphere. All comparisons are relative to NC cohort. *Top row:* AD vs. NC; *Middle:* MCI subjects showing AD atrophy pattern vs. NC; *Bottom:* MCI subjects without AD atrophy pattern vs. NC. *LEFT* mesial views, *RIGHT* lateral views. The scale ranges from $<$–0.3 (yellow) to $> +0.3$ (cyan) mm thickness. Areas on the red-yellow spectrum indicate regions of thinning with disease.

## 10. Classification of MCI subgroups

This discriminant model was then applied to MCI data, producing an "atrophy score" for each MCI individual reflecting the degree to which the individual's MRI reflected the presence of characteristic AD atrophy. On the basis of this score, each MCI participant was classified as showing either the NC or AD "imaging phenotype" (Fig. 3). MCI participants with AD atrophy showed an imaging pattern nearly identical to that of the AD group, with structural thinning evident even in regions not contributing to the atrophy score. MCI subjects without the AD atrophy pattern displayed less widespread atrophy, with a reduced level of atrophy in hippocampal and middle temporal regions. MCI individuals with AD atrophy had higher baseline CDR sum of boxes and lower verbal memory scores but equivalent MMSE scores at baseline.

In essence, the pattern that best discriminated NC from AD, and that was predictive of decline in MCI, involved the mesial temporal, lateral temporal, retrosplenial and orbitofrontal regions. Mesial temporal structures have long been implicated early in AD, and atrophy in these structures has been found to be predictive of disease progression [21,22,44,58,79]. Lateral temporal areas, including inferior, middle, and superior temporal gyri, have been implicated in the progression of AD [13,72,86,87], and we demonstrated in our morphometric study that there is also evidence of such changes in SMCI [29]. These findings support a pattern of preclinical cortical atrophy that may be predictive of a more rapid course of disease progression.

## 11. Prediction of clinical decline

Of significant interest, follow-up data were available for 160 of these MCI cases for a year from baseline evaluation. Those expressing the baseline AD phenotype showed a significant decline in MMSE score over one year, whereas those with the NC phenotype remained stable. The atrophy score was the primary predictor for this decline, although additional power in prediction came from Apolipoprotein E (APOE) $\varepsilon 4$ allele status (a genetic risk factor for late-onset AD [6]) and Logical Memory Delayed Recall scores. Interestingly, four MCI individuals reverted to normal cognitive status (three showed the NC imaging phenotype) and 33 converted to a diagnosis of AD (23 showed the AD phenotype). Those who declined in MMSE score over one year were significantly more likely to show the AD phenotype, the conversion rate among MCI subjects with the AD phenotype was 32% relative to only 11% for those with the NC phenotype. This contrasts with a recent report that indicated that whole brain, ventricular, entorhinal and hippocampal volumes did not provide additional predictive information of clinical decline beyond that attainable with clinical measures [34], and shows the value of examining individually specific brain regions beyond the mesial temporal lobe.

## 12. Summary

In summary, the literature supports the strong potential of structural MRI profiles, even within cross-sectional designs, as a promising method for the discrimination of AD from normal controls and in the prediction of MCI progression and conversion to AD. Findings suggest that measures of structural change in mesial and lateral temporal, cingulate, parietal and mid-frontal areas may facilitate the assessment of a treatment's ability to halt the progressive structural loss that accompanies clinical decline in MCI. Of course, there remains significant potential to improve the performance of prediction. For example, using follow-up data from ADNI's large MCI cohort to identify structural patterns associated with MCI individuals who convert to AD relative to those who do not [62] may better identify the structural changes that are predictive of disease progression. In addition, the incorporation of measures from other neuroimaging modalities, clinical assessments, and neuromedical biomarkers, as being assessed in current work (e.g. [63,74]), may also help to refine the regional profile of individuals at risk for progression.

## Acknowledgments

This work was supported by grants from the National Center for Research Resources (U24 RR021382 – CFN, AMD; DJH); the National Institute on Aging (R01AG22381 – CFN, LKM, AMD; K01AG029218 – LKM); and the Department of Veterans Affairs (Advanced Career Dev. Award – MWJ). This review refers to important work facilitated by the Alzheimer's Disease Neuroimaging Initiative (ADNI; Principal Investigator: Michael Weiner; NIH grant U01 AG024904), which funded data collection and sharing. ADNI is funded by the National Institute on Aging, the National Institute of Biomedical Imaging and Bioengineering (NIBIB), and through generous contributions from the following: Pfizer Inc., Wyeth Research, Bristol-Myers Squibb, Eli Lilly and Company, GlaxoSmithKline, Merck & Co. Inc., AstraZeneca AB, Novartis Pharmaceuticals Corporation, Alzheimer's Association, Eisai Global Clinical Development, Elan Corporation plc, Forest Laboratories, and the Institute for the Study of Aging, with participation from the U.S. Food and Drug Administration. Industry partnerships are coordinated through the Foundation for the National Institutes of Health. The grantee organization is the Northern California Institute for Research and Education, and the study is coordinated by the Alzheimer's Disease Cooperative Study at the University of California, San Diego. ADNI data are disseminated by the Laboratory of Neuro Imaging at the University of California, Los Angeles.
